# Exercise Training Protects against Atorvastatin-Induced Skeletal Muscle Dysfunction and Mitochondrial Dysfunction in the Skeletal Muscle of Rats

**DOI:** 10.3390/jcm9072292

**Published:** 2020-07-19

**Authors:** Dae Yun Seo, Jun-Won Heo, Mi-Hyun No, Su-Zi Yoo, Jeong Rim Ko, Dong-Ho Park, Ju-Hee Kang, Chang-Ju Kim, Su-Jeen Jung, Jin Han, Hyo-Bum Kwak

**Affiliations:** 1 Department of Physiology, National Research Laboratory for Mitochondrial Signaling, BK21 Plus Project Team, College of Medicine, Smart Marine Therapeutics Center, Cardiovascular and Metabolic Disease Center, Inje University, Busan 47392, Korea; sdy925@gmail.com (D.Y.S.); kjrsos0217@gmail.com (J.R.K.); 2 Department of Kinesiology, Inha University, Incheon 22212, Korea; gjwnsdnjs03@naver.com (J.-W.H.); 77nodaji@hanmail.net (M.-H.N.); susie_737@naver.com (S.-Z.Y.); dparkosu@inha.ac.kr (D.-H.P.); 3 Department of Pharmacology and Medicinal Toxicology Research Center, Inha University School of Medicine, Incheon 22212, Korea; johykang@inha.ac.kr; 4 Department of Physiology, College of Medicine, Kyung Hee University, Seoul 02447, Korea; changju@khu.ac.kr; 5 Department of Leisure Sports, Seoil University, Seoul 02192, Korea; sujeenj@nate.com

**Keywords:** statin, exercise, myopathy, mitochondria, skeletal muscle

## Abstract

Statins are used to prevent and treat atherosclerotic cardiovascular disease, but they also induce myopathy and mitochondrial dysfunction. Here, we investigated whether exercise training prevents glucose intolerance, muscle impairment, and mitochondrial dysfunction in the skeletal muscles of Wistar rats treated with atorvastatin (5 mg kg^−1^ day^−1^) for 12 weeks. The rats were assigned to the following three groups: the control (CON), atorvastatin-treated (ATO), and ATO plus aerobic exercise training groups (ATO+EXE). The ATO+EXE group exhibited higher glucose tolerance and forelimb strength and lower creatine kinase levels than the other groups. Mitochondrial respiratory and Ca^2+^ retention capacity was significantly lower in the ATO group than in the other groups, but exercise training protected against atorvastatin-induced impairment in both the soleus and white gastrocnemius muscles. The mitochondrial H_2_O_2_ emission rate was relatively higher in the ATO group and lower in the ATO+EXE group, in both the soleus and white gastrocnemius muscles, than in the CON group. In the soleus muscle, the Bcl-2, SOD1, SOD2, Akt, and AMPK phosphorylation levels were significantly higher in the ATO+EXE group than in the ATO group. In the white gastrocnemius muscle, the SOD2, Akt, and AMPK phosphorylation levels were significantly higher in the ATO+EXE group than in the ATO group. Therefore, exercise training might regulate atorvastatin-induced muscle damage, muscle fatigue, and mitochondrial dysfunction in the skeletal muscles.

## 1. Introduction

Statins are a major group of lipid-lowering drugs that inhibit 3-hydroxy-3-methylglutaryl coenzyme A reductase [1,2]. They are used in clinical trials to prevent atherosclerotic cardiovascular disease [3]. There is a growing body of literature that recognizes the importance of statins for patients older than 65 years with cardiovascular disease, who account for approximately 25% of the world’s population [4,5]. This suggests that the number of patients treated with statins will continue to increase; therefore, clinical research into the use of statins must be carried out.

Statin treatment is relatively safe for patients with dyslipidemia and cardiovascular diseases [6,7,8]. Specifically, statin treatment often causes adverse skeletal muscle symptoms, including skeletal muscle pain, weakness, and fatal rhabdomyolysis [9], which are associated with major pathological changes, such as the elevation of creatine kinase (CK) levels [10], reduction of muscular strength, muscle weakness [11], induction of apoptosis [12], and mitochondrial dysfunction [13] in skeletal muscles. In contrast, Tinna et al. [14] found no change in muscular function following statin therapy. Atorvastatin is one of the most effective statins, considering not only its effects on low-density lipoprotein-cholesterol and its capability of meeting recommended therapy guidelines for this parameter but also its effect on triglyceride (TG) levels and its capacity to change lipoprotein composition in a non-atherogenic manner [15]. Despite the well-known positive and negative physiological effects, the exact mechanisms involved in the development of statin-induced myopathy remain unclear.

Exercise training plays an important role in physiological adaptation, improves whole-body glucose metabolism [16,17], skeletal muscle metabolism [18,19], and mitochondrial function [20,21] and prevents apoptosis [22]. A recent study has revealed that simvastatin treatment plus 12 weeks of exercise attenuates the increase in skeletal muscle mitochondrial contents when combined with 12 weeks of exercise training in sedentary, overweight, and obese adults [23]. Liliana et al. [24] suggested that statins, combined with exercise training, improve body weight, glucose homeostasis, skeletal muscle strength, and lipid profile. Conversely, a daily dose of 40 mg lovastatin increased the CK levels after exercise [25], and Boston marathoners treated with statins show higher CK levels than those who did not receive statins [26]. Therefore, the aim of this study was to examine whether exercise training attenuates atorvastatin-induced myopathy and mitochondrial dysfunction in the skeletal muscles. We hypothesized that exercise training would attenuate atorvastatin-induced skeletal muscle fatigue, atrophy, and mitochondrial dysfunction in rats.

## 2. Materials and Methods

### 2.1. Animal Experiments

Seven-week-old, male Wistar rats were obtained from Orient Bio Company (Seongnam-si, Gyeonggi-do, Korea). The animals were maintained at 20 ± 2.5 °C under a 12-h light–dark cycle, with free access to food and water. After 1 week of acclimation, 36 rats were randomly assigned to three groups, namely the water-treated (CON, *n* = 12), atorvastatin-treated (ATO, *n* = 12), and ATO plus aerobic exercise training group (ATO+EXE, *n* = 12). All experimental procedures were approved by the Institutional Animal Use and Care Committee of Kyung Hee University (KHUASP(SE)-16-064).

### 2.2. Atorvastatin Treatment

Atorvastatin (Tahor^®®^) was obtained from Pfizer (New York, NY, USA). The treatments were administered via oral gavage. The vehicle-treated rats received water in 0.25% w/v hydroxypropyl methylcellulose (HPMC), whereas the atorvastatin-treated rats received atorvastatin (5 mg kg^−1^ day^−1^) dissolved in 0.25% w/v HPMC, for 12 weeks.

### 2.3. Exercise Training

The ATO+EXE group was trained for 12 weeks on a 15% incline, 20 m min^−1^ for 60 min day^−1^, 5 days per week^−1^, using a rat treadmill (Eco 3/6 treadmill; Columbus Instruments, Columbus, OH, USA), as previously reported [27,28].

### 2.4. Forelimb Grip Strength Test

After 12 weeks, a forelimb grip strength test was performed using an automated grip strength meter (Columbus Instruments). The rats were grasped by the bottom of the tail and hung onto the grip ring. After approximately 3 s, the rats were gently pulled towards the grip ring until they released their grip. The average grip strength force in grams was determined using a computerized electronic strain gauge mounted directly on the grip ring. We calculated the maximal forelimb strength from the average of three best trials, as previously reported [29]. In addition, the fatigue index was measured by calculating the degree of fatigue by comparing the first two pulls to the last two pulls. Both values were normalized by body weight.

### 2.5. Oral Glucose Tolerance Test

To measure the blood glucose level, which reflects insulin resistance, an oral glucose tolerance test (OGTT) was performed in all the groups after 12 weeks. Prior to OGTT, the rats fasted for 12 h and were orally administered a glucose solution (1.5 g kg^−1^ dissolved in sterile saline) by oral gavage. Blood samples were collected from the tail vein and the blood glucose level was measured before oral administration (0 min) and at 30, 60, and 120 min following oral administration, using an SD Code-Free blood glucose meter (SD BIOSENSOR, Inc., Suwon, Korea).

### 2.6. Western Blotting

The soleus and white gastrocnemius muscles were homogenized in ice-cold lysis buffer (50 mM HEPES, 10 mM EDTA, 100 mM NaF, 50 mM Na pyrophosphate, 10 mM Na orthovanadate, and 1% Triton at pH 7.4), supplemented with protease/phosphatase inhibitor cocktails (Thermo Fisher Scientific, Waltham, MA, USA), using a Polytron homogenizer for 30 s. The protein concentration in the tissue lysates was determined using the bicinchoninic acid assay method. To evaluate the levels of Bax, Bcl-2, cleaved caspase-3, SOD1, SOD2, *p*-Akt, t-Akt, *p*-AMPK, t-AMPK, and beta actin in the skeletal muscle, 30 μg of the lysates was subjected to SDS-PAGE and then transferred onto polyvinyldifluoride membranes (Millipore, Bedford, MD, USA). All primary antibodies were diluted to a concentration of 1:1000. The blots were detected using a ChemiDoc™ XRS + System (Bio-Rad Laboratories, Inc., Hercules, CA, USA) and band densities were quantified using Image Lab™ software (Bio-Rad Laboratories, Inc., Hercules, CA, USA).

### 2.7. Serum Creatine Kinase Measurement

The rats were fully anesthetized with ethyl ether and euthanized by cardiac puncture. Blood was obtained, evaluated using the colorimetric Serum Creatine Kinase Assay Kit (Abcam, Cambridge, MA, USA), and calculated using a spectrophotometer.

### 2.8. Tissue Preparation

The soleus (type I fiber) and white gastrocnemius (type IIb fiber) muscles were dissected and removed. Then, the connective tissues were removed and the muscles were rinsed in PBS, dried, weighed, frozen in liquid nitrogen, and stored at −80 °C until analysis.

### 2.9. Hematoxylin-Eosin Stain

The soleus (type I fiber) and plantaris (type II fiber) muscles were fixed in 4% paraformaldehyde for 24 h. The fixed tissues were dehydrated, treated with xylenes, embedded, and cut into 5 µm sections, according to the standard procedures [30]. The sections were stained with hematoxylin-eosin stain and images were captured using a digital camera (Olympus Corporation, Tokyo, Japan). The cross-section area and endomysium space were calculated using Image J software [10].

### 2.10. Preparation of Permeabilized Muscle Fiber Bundles

Following dissection of the soleus and white gastrocnemius, a pair of micro-forceps and a dissecting microscope were used to separate the skeletal muscles in ice-cold buffer X containing 7.23 mM K_2_EGTA, 2.77 mM Ca K_2_EGTA, 20 mM imidazole, 0.5 mM DTT, 20 mM taurine, 5.7 mM ATP, 14.3 mM phosphocreatine, 6.56 mM MgCl_2_-6H_2_O, and 50 mM MES (pH 7.1, 290 mOsm). The separated myofiber (approximately 2 mg) was permeabilized with saponin (50 µg mL^−1^). Saponin is a mild, cholesterol-specific detergent that selectively permeabilizes cholesterol-rich sarcolemmal membranes, keeping the mitochondrial membrane intact because the mitochondrial membranes do not have significant levels of cholesterol. After permeabilization for 30 min at 4 °C, the permeabilized myofiber was washed with washing buffer (buffer Z with 50 μM EGTA) containing 105 mM K-MES, 30 mM KCl, 10 mM KH_2_PO_4_, 5 mM MgCl_2_-6H_2_O, and 0.5 mg mL^−1^ BSA (pH 7.1) for at least 15 min, retained in the washing buffer, and placed in a shaker, at 4 °C, until further analysis. This permeabilized myofiber was used to measure mitochondrial oxygen (O_2_) respiration, hydrogen peroxide (H_2_O_2_) emission, and Ca^2+^ retention capacity.

### 2.11. Mitochondrial O_2_ Respiration

As previously described [31], mitochondrial O_2_ respiration was measured using a polarographic high-resolution respirometer (O_2_K Oxygraph, Oroboros, Innsbruck, Austria) at 30 °C in an assay respiration buffer (Buffer Z with 20 mM creatine and 50 µM EGTA) using the following protocol: (i) 5 mM glutamate (complex I substrate) + 2 mM malate (complex I substrate) and (ii) 4 mM ADP (state 3 condition). After completion of the experiment, the mitochondrial oxygen respiration level was normalized using the wet weight of the tissue. The mitochondrial O_2_ respiration is expressed as picomoles s^−1^ mg^−1^ wet tissue weight.

### 2.12. Mitochondrial H_2_O_2_ Emission

After separation, permeabilization, and washing of the skeletal muscle tissue, mitochondrial H_2_O_2_ emission was measured in buffer Z at 37 °C (ΔF min^−1^) during state 4 respiration (10 µg mL^−1^ oligomycin) by continuously monitoring the oxidation of Amplex^®®^ Red (excitation/emission wavelengths λ = 568/581 nm) using a SPEX Fluormax 4 spectrofluorometer (HORIBA Jobin Yvon, Edison, NJ, USA), using the following protocol: approximately 2 mg muscle fiber sample, 10 µM Amplex^®®^ Red, 1.5 U mL^−1^ horseradish peroxidase, 10 µg mL^−1^ oligomycin settings, and (i) 5 mM glutamate (complex I substrate) + 2 mM malate (complex I substrate), (ii) 10 mM succinate (complex II substrate), and (iii) 10 mM glycerol-3 phosphate (lipid substrate). After removing the background value from each of the standard values (standard curve), the mitochondrial H_2_O_2_ emission rate was calculated from the slope of ΔF min^−1^ gradient values. After completion of the experiment, the H_2_O_2_ emission level was normalized using the wet weight of the tissue. The rate of mitochondrial H_2_O_2_ emission is expressed as picomoles min^−1^ mg^−1^ wet tissue weight.

### 2.13. Mitochondrial Ca^2+^ Retention Capacity

To evaluate the susceptibility of the permeability transition pore (PTP) to opening, mitochondrial Ca^2+^ retention capacity was determined as previously reported but with some modifications [30]. Briefly, following separation, permeabilization, and washing of the skeletal muscle tissue, overlaid traces of changes in Ca^2+^-induced fluorescence by Calcium Green-5 N were measured continuously (ΔF / min) at 37 °C during state respiration 4, using the Spex Fluormax 4 spectrofluorometer (HORIBA Jobin Yvon, Edison, NJ, USA). After establishing the background ΔF (1 μM Calcium Green-5 N, 80 μM EGTA, 5 mM glutamate, and 2 mM malate in buffer Z), the reaction was initiated by the addition of Ca^2+^ pulses (30 μM), with excitation and emission wavelengths set at 506 and 532 nm, respectively. After completion of the experiment, mitochondrial Ca^2+^ retention capacity was normalized using the wet weight of the tissue (picomoles mg^−1^). Aggregate mitochondrial Ca^2+^ retention capacity before PTP opening (i.e., Ca^2+^ release from mitochondria) is expressed as picomoles mg^−1^ wet tissue weight.

### 2.14. Statistical Analysis

All data are presented as means ± standard error of mean (SEM). The statistical analyses were performed using SPSS 25 (SPSS, Inc., Chicago, IL, USA) and differences among groups were determined using a one-way ANOVA with Tukey’ post hoc test. The level of statistical significance was set at *p* < 0.05.

## 3. Results

### 3.1. Exercise Training Prevents Skeletal Muscle Fatigue in the Skeletal Muscles of Rats Treated with Atorvastatin

**Figure 1:**
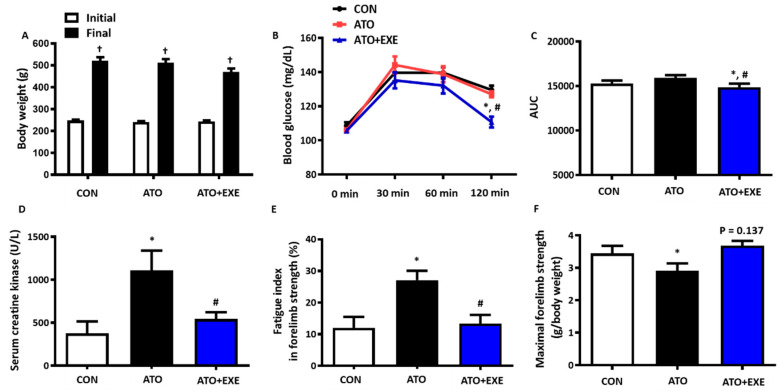
Exercise training prevents skeletal muscle fatigue in the skeletal muscles of rats treated with atorvastatin. (**A**) Body weight; (**B**) Blood glucose; (**C**) Area under the curve (AUC); (**D**) Serum creatine kinase; (**E**) Fatigue index in forelimb strength; (**F**) Maximal forelimb strength. Data are presented as means ± SEM. ^†^ *p* < 0.05 vs. initial; * *p* < 0.05 vs. control (CON); ^#^ *p* < 0.05 vs. atorvastatin-treated (ATO).

In all groups, the final body weight of rats was significantly higher than the initial weight. The ATO+EXE group had no effect on body weight (Figure 1A). We tested whether exercise training attenuated the susceptibility to atorvastatin-induced glucose intolerance. Blood glucose levels were significantly lower in the ATO+EXE group than in the other groups throughout the 120 min duration of the test and the area under the curve of blood glucose response (*p* < 0.05; Figure 1B,C). Serum CK levels and fatigue index were significantly higher in the ATO group than in the CON group, whereas ATO+EXE attenuated these markers (*p* < 0.05; Figure 1D,E). In addition, maximal forelimb strength was significantly lower in the ATO group than in the CON group (*p* < 0.05; Figure 1F). Although a tendency towards an increase in maximal forelimb strength was observed in ATO+EXE group, statistical significance was not reached (*p* = 0.137; Figure 1F)

### 3.2. Atorvastatin Induces Skeletal Muscle Atrophy

**Figure 2:**
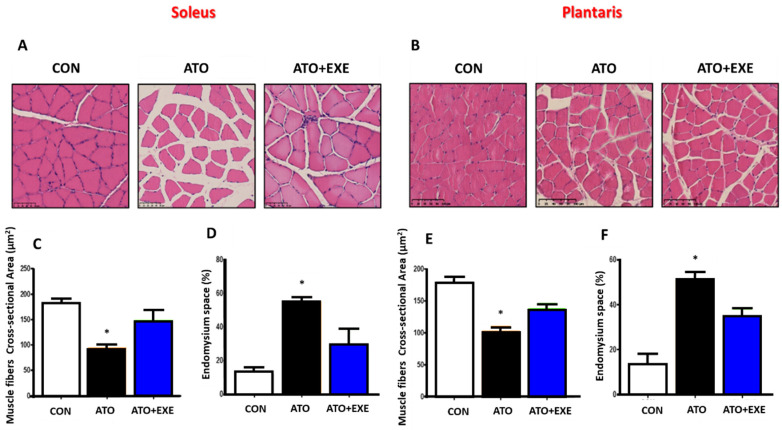
Atorvastatin induces skeletal muscle atrophy. Representative hematoxylin-eosin stained soleus (**A**) and plantaris (**B**) from rats; muscle fiber cross-sectional area in soleus (**C**); endomysium space in soleus (**D**); muscle fiber cross-section area in plantaris (**E**); endomysium space in plantaris (**F**). Scale bars, 100 µm. Data are presented as means ± SEM. * *p* < 0.05 vs. CON.

The muscle fiber cross-sectional area and endomysium space were evaluated in the soleus and plantaris muscles (Figure 2A,B). In both muscles, the muscle fiber cross-sectional area was significantly lower in the ATO group than in the CON group (*p* < 0.05; Figure 2C,E). However, there was no significant difference between ATO and ATO+EXE. In both soleus and plantaris muscles, the endomysium space was significantly larger in the ATO group than in the CON group (*p* < 0.05; Figure 2D,F), whereas there was no significant difference between the ATO and ATO+EXE groups.

### 3.3. Exercise Training Improves Mitochondrial O_2_ Respiration in the Skeletal Muscles of Rats Treated with Atorvastatin

**Figure 3:**
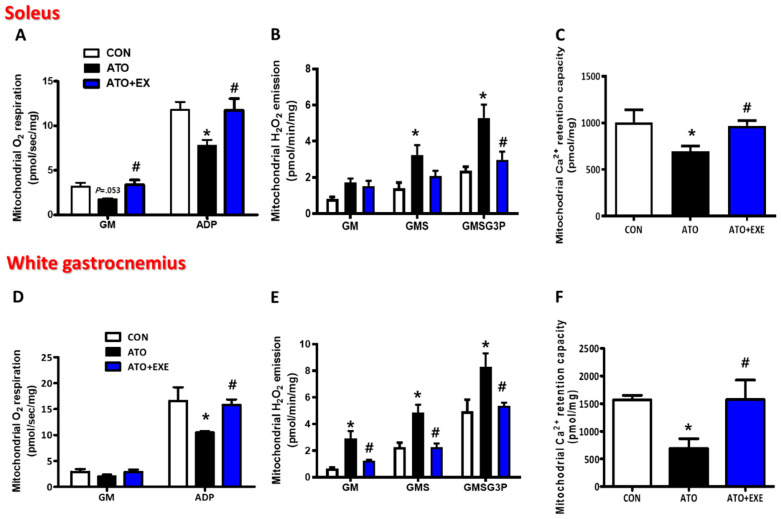
Exercise improves mitochondrial O_2_ respiration, H_2_O_2_ emission, and Ca^2+^ retention capacity in the skeletal muscles of rats treated with atorvastatin. (**A**) O_2_ respiration in the soleus; (**B**) H_2_O_2_ emission in the soleus; (**C**) Ca^2+^ retention capacity in the soleus; (**D**) O_2_ respiration in the white gastrocnemius; (**E**) H_2_O_2_ emission in the white gastrocnemius; (**F**) Ca^2+^ retention capacity in the white gastrocnemius. Data are presented as means ± SEM. * *p* < 0.05 vs. CON; ^#^ *p* < 0.05 ATO.

The mitochondrial O_2_ respiration protocol was based on glutamate-malate-supported respiration (electron entry via complex I, state 2) and ADP (state 3) (Figure 3). In the soleus, the basal respiration (state 2) supported by glutamate-malate (GM, substrates of complex I, state 2) did not differ significantly between the ATO and CON groups (3.18 ± 0.43 vs. 1.77 ± 0.09, *p* = 0.053; Figure 3A). However, in maximal ADP-stimulated O_2_ respiration (state 3) supported by glutamate-malate (ADP), mitochondrial O_2_ consumption was significantly lower (by 35%) in the permeabilized myofibers of ATO than in normal permeabilized myofibers (11.78 ± 7.55 vs. 7.76 ± 0.68, *p* < 0.05; Figure 3A). Similar to the soleus muscle, in the white gastrocnemius, mitochondrial O_2_ consumption supported by GM did not differ significantly between the ATO and CON groups (2.88 ± 0.57 vs. 2.05 ± 0.36; Figure 3D). Moreover, consistent with the soleus, maximal ADP-stimulated O_2_ consumption (state 3) was significantly lower (by 37%) in the ATO group than in the CON group (16.6 ± 2.63 vs. 10.56 ± 0.23, *p* < 0.05; Figure 3D). Mitochondrial O_2_ respiration was significantly higher in the ATO+EXE group than in the ATO group. Specifically, in the soleus muscles, mitochondrial O_2_ respiration was higher in all stages, including basal respiration and maximal ADP-stimulated respiration (1.77 ± 0.09 vs. 3.39 ± 0.51, 7.76 ± 0.68 vs. 11.75 ± 1.30, respectively, *p* < 0.05; Figure 3A), and, in white gastrocnemius muscles, although there was no difference in GM-supported respiration, mitochondrial ADP-stimulated O_2_ consumption was significantly higher (2.05 ± 0.36 vs. 2.88 ± 0.57, 10.56 ± 0.23 vs. 15.81 ±1.08, *p* < 0.05; Figure 3D) in the ATO+EXE group than in the ATO group.

### 3.4. Exercise Improves Mitochondrial H_2_O_2_ Emission and Ca^2+^ Retention Capacity in the Skeletal Muscles of Rats Treated with Atorvastatin

The mitochondrial H_2_O_2_ emission protocol was based on GM (complex I substrates), succinate (GMS, complex II substrate), and glycerol-3-phosphate (GMSG3P, lipid substrate) (Figure 3B,E). In the soleus muscles, the mitochondrial H_2_O_2_ emission was not altered in the ATO group in basal conditions supported by GM (complex I substrates). In the next stage, GMS (complex II substrates) supported, mitochondrial H_2_O_2_ emission was 130% higher in the ATO group than in the CON group (1.40 ± 0.31 vs. 3.24 ± 0.55, *p* < 0.05; Figure 3B). Furthermore, with GMSG3P (lipid substrates), the ATO group had excessive H_2_O_2_ emission, being 122% higher than that in the CON group (2.38 ± 0.21 vs. 5.29 ± 0.74, *p* < 0.05; Figure 3B). Unlike the soleus muscles, in the white gastrocnemius, an atorvastatin-induced increase in mitochondrial H_2_O_2_ emission was found in all stages, including GM, GMS, and GMSG3P (*p* < 0.05; Figure 3E). However, the excessively emitted mitochondrial H_2_O_2_ in the ATO group was significantly attenuated in the ATO+EXE group (*p* < 0.05; Figure 3E). In the soleus muscles, excessive mitochondrial H_2_O_2_ emission was only found at the GMSG3P stage, being 44% higher in the ATO group than in the ATO+EXE group (5.29 ± 0.74 vs. 2.98 ± 0.45, *p* < 0.05; Figure 3B). Moreover, in the white gastrocnemius muscles, the excessive mitochondrial H_2_O_2_ emission in the ATO group was reduced at all stages (GM, GMS, and GMSG3P) in the ATO+EXE group. The mitochondrial Ca^2+^ retention capacity in the permeabilized myofibers was significantly lower in the ATO group than in the CON group in the soleus (31% lower) and white gastrocnemius (57% lower) muscles (1007.26 ± 134.59 vs. 697.05 ± 54.98, 1570.48 ± 78.48 vs. 690.49 ± 174.92, respectively, *p* < 0.05; Figure 3C,F). However, the ATO+EXE group had a higher mitochondrial Ca^2+^ retention capacity in the soleus and white gastrocnemius muscles than the ATO group (697.05 ± 54.98 vs. 968.88 ± 57.34, 690.49 ± 174.92 vs. 1578.29 ± 346.10, *p* < 0.05; Figure 3C,F), which demonstrated that exercise training plays an important role in attenuating decreases in mitochondrial Ca^2+^ retention capacity in skeletal muscles owing to atorvastatin treatment.

### 3.5. Effects of Exercise Training on Mitochondria-Mediated Apoptotic Signaling in the Skeletal Muscles of Rats Treated with Atorvastatin

**Figure 4:**
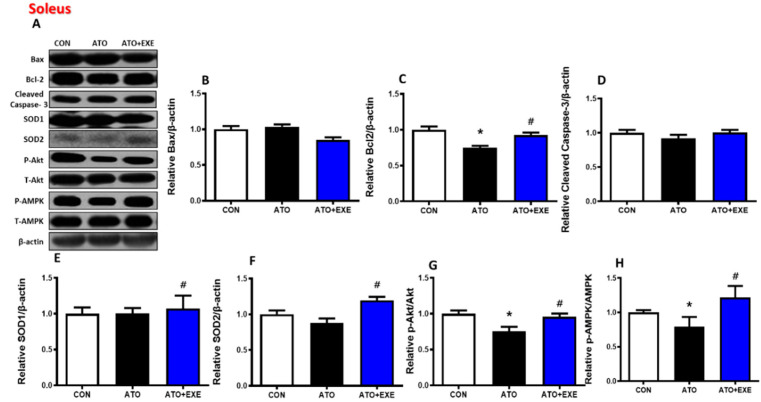
Effects of exercise training on mitochondria-mediated apoptotic signaling in the skeletal muscles of rats treated with atorvastatin. Quantification of Western blots normalized to β-actin. (**A**) bands; (**B**) Bax; (**C**) Bcl-2; (**D**) cleaved caspase-3; (**E**) SOD1; (**F**) SOD2; (**G**) *p*-Akt/t-Akt; (**H**) *p*-AMPK/t-AMPK. Data are presented as means ± SEM. * *p* < 0.05 vs. CON; ^#^ *p* < 0.05 ATO.

**Figure 5:**
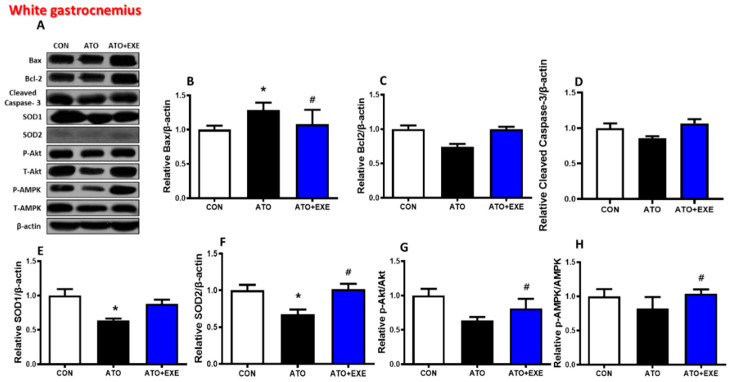
Effects of exercise training on mitochondria-mediated apoptotic signaling in the skeletal muscles of rats treated with atorvastatin. Quantification of Western blots normalized to β-actin. (**A**) bands; (**B**) Bax; (**C**) Bcl-2; (**D**) cleaved caspase-3; (**E**) SOD1; (**F**) SOD2; (**G**) *p*-Akt/t-Akt; (**H**) *p*-AMPK/t-AMPK. Data are presented as means ± SEM. * *p* < 0.05 vs. CON; ^#^ *p* < 0.05 ATO.

In the soleus, Bax and cleaved caspase-3 protein levels did not change in the ATO and ATO+EXE groups (Figure 4B,D). The levels of Bcl-2, Akt, and AMPK phosphorylation were significantly lower in the ATO group than in the CON group, whereas they were significantly higher in the ATO+EXE group (*p* < 0.05; Figure 4C,G, and H). The levels of SOD1 and SOD2 were significantly higher in the ATO+EXE group than in the ATO group (*p* < 0.05; Figure 4E,F). In the white gastrocnemius, Bax protein levels were significantly higher in the ATO group than in the CON group. However, exercise training decreased Bax protein levels (*p* < 0.05; Figure 5B). Bcl-2 and cleaved capase-3 protein levels did not change in the ATO and ATO+EXE groups (Figure 5C,D). SOD1 and SOD2 levels were significantly lower in the ATO group than in the CON group (*p* < 0.05; Figure 5E,F). However, the levels of SOD2, Akt, and AMPK phosphorylation were significantly higher in the ATO+EXE group than in the ATO group (*p* < 0.05; Figure 5F–H).

## 4. Discussion

Here, we provide evidence that (1) exercise training attenuated atorvastatin-induced glucose intolerance and increased serum CK level and fatigue index; (2) atorvastatin treatment decreased muscle fiber cross-sectional area and increased endomysium space in both soleus and white gastrocnemius muscles, but exercise training did not completely recover these markers; (3) maximal ADP-stimulated O_2_ respiration decreased in atorvastatin-treated rats but improved with exercise training in both soleus and white gastrocnemius myofibers; (4) atorvastatin treatment decreased the protein levels of Bcl-2, Akt, and AMPK phosphorylation, but exercise training recovered these markers and increased the levels of SOD1 and SOD2 proteins in the soleus; (5) atorvastatin treatment decreased the protein levels of Bcl-2 and SOD2, but exercise training recovered these markers and increased the protein levels of SOD1, Akt, and AMPK phosphorylation in the white gastrocnemius. These findings support our hypothesis that atorvastatin-induced myopathy is inhibited by exercise training. Indeed, exercise training plays a critical role in preventing atorvastatin-induced skeletal myopathy and mitochondrial dysfunction in skeletal muscles.

Skeletal muscle damage is associated with the elevation of serum CK levels, regulation of skeletal muscle dysfunction, and fatigue [32]. Atorvastatin treatment elevates serum CK levels, leads to muscle fatigue, reduces muscular strength, and induces mitochondrial dysfunction [33,34,35,36]. We found that atorvastatin induced an elevation in serum CK level and fatigue index and reduced maximal forelimb strength (Figure 1). These results are consistent with previous findings which indicate that two weeks of statin treatment impairs skeletal muscle function, including reduced grip strength, maximal force, and fatigue [37,38]. In addition, we found that the skeletal muscles of rats treated with atorvastatin had a decreased muscle fiber cross-sectional area and an increased endomysium space (Figure 2). These results show that rats treated with atorvastatin had increased skeletal muscle dysfunction, resulting in skeletal muscle atrophy [39,40]. However, exercise training did not improve the muscle fiber cross-sectional area and endomysium space, although there was a tendency towards an increase in muscle fiber cross-sectional area and a decrease in endomysium space with 12 weeks of exercise training. These findings provide evidence that a 12-week aerobic exercise training program may not be of a sufficient duration to induce a recovery of morphological changes in skeletal muscle. Importantly, exercise training attenuated the increase in serum CK levels and fatigue index, which is consistent with the results of previous studies [25,41]. Therefore, further study is necessary to elucidate the influence of exercise types and duration on the muscle fiber cross-sectional area and endomysium space in skeletal muscle of rats treated with atorvastatin.

Atorvastatin also stimulates mitochondrial dysfunction [34,42]. Interestingly, exercise training exacerbates atorvastatin-induced mitochondrial dysfunction in skeletal muscles [43,44]. Based on previous studies, we investigated whether mitochondrial function (e.g., O_2_ respiration, H_2_O_2_ emission, and Ca^2+^ retention capacity) was affected by in vivo atorvastatin treatment and aerobic exercise training in permeabilized myofibers. In the current study, we demonstrated that atorvastatin-induced mitochondrial dysfunction was mitigated by exercise training. Specifically, damaged mitochondrial O_2_ respiration was observed in the ATO group under maximal respiration conditions stimulated by ADP (state 3) (Figure 3A,D), suggesting that, as O_2_ consumption in the mitochondria and ATP production are simultaneously coupled, energy metabolism by the electron transfer chain in mitochondria may be dysregulated by atorvastatin treatment. We also analyzed mitochondrial H_2_O_2_ emission. Physiologically, appropriate H_2_O_2_ production by the mitochondria plays a pivotal role in regulating cellular redox conditions and mitochondrial function [45,46]. However, pathologically, excessive reactive oxygen species production can damage the cell and mitochondria [47]. Indeed, studies show that H_2_O_2_ production is elevated in atorvastatin-treated skeletal muscles [31,48]. Consistent with these studies, excessive mitochondrial H_2_O_2_ emission was found in the ATO group compared with the CON group at all stages (GM, GMS, GMSG3P; Figure 3B,E), suggesting that excessive mitochondrial H_2_O_2_ emission, induced by atorvastatin treatment, may lead to mitochondrial DNA mutation [49], damaged mitochondrial structure, and apoptosis [50]. Although we did not directly measure whether these levels of mitochondrial H_2_O_2_ emission were appropriate or excessive, we suspect that excessive H_2_O_2_ was generated by mitochondria in the ATO group, owing to the significant difference among the groups. In addition, mitochondrial Ca^2+^ retention capacity, which is a threshold of Ca^2+^ overload-induced mitochondrial permeability transition pore (mPTP) opening, was lower in atorvastatin-treated skeletal muscles than in normal skeletal muscles. Mitochondrial Ca^2+^ regulates the energy production in the tricarboxylic acid cycle by controlling the mitochondrial enzymes [51,52] and is associated with apoptosis, which results from mitochondrial Ca^2+^ overload. Previous studies show that statin treatment increases Ca^2+^-induced mPTP opening in isolated rat skeletal muscles [53]. Consistent with previous studies, we demonstrated that mitochondrial Ca^2+^ retention capacity was lower in the ATO group than in the CON group, as demonstrated by the significantly lower mitochondrial Ca^2+^ retention capacity in the ATO group (Figure 3C,F). Consequently, atorvastatin may induce mitochondria-mediated apoptosis from the mitochondrial intermembrane to the cytosol (Figure 4 and Figure 5).

In this study, morphological changes, mitochondrial dysfunction, and mitochondria-mediated apoptotic signaling by atorvastatin were not significantly different between type I and type II fibers, which is inconsistent with previous study [12]. According to one previous study, atorvastatin treatment for 2 weeks at 10 mg kg^−1^ per day^−1^ in rats increases H_2_O_2_ accumulation, mRNA levels, and the Bax/Bcl-2 ratio, as well as TUNEL staining and caspase-3 cleavage, in type II glycolytic (plantaris) skeletal muscle but not in type I oxidative (soleus) skeletal muscle, which has a high antioxidative capacity [12]. Glycolytic muscles are more sensitive to atorvastatin than oxidative muscles, which may be owing to the higher antioxidative capacity of oxidative muscles.

Exercise training plays an important role in the alleviation of mitochondrial dysfunction in atorvastatin-induced skeletal muscle myopathy. Mitochondrial dysfunction was observed in atorvastatin-treated skeletal muscles in the ATO group; however, exercise training attenuated this in permeabilized myofibers. Firstly, mitochondrial O_2_ respiration was higher in the ATO+EXE group than in the ATO group. Importantly, mitochondrial O_2_ respiration in the state 3 condition (GM3) was improved by exercise training (Figure 3A,D), indirectly suggesting that exercise training improves ATP production coupled with oxygen consumption in the mitochondria. Secondly, excessive H_2_O_2_ emission generated by the mitochondria was down-regulated by exercise training in the ATO+EXE group in all stages, suggesting that the exercise training attenuates mitochondrial damage by atorvastatin treatment. Finally, exercise training increased the mitochondrial Ca^2+^ retention capacity, which is reduced by atorvastatin treatment (Figure 3B,C,E,F), confirming the results of previous studies [41,44]. Therefore, exercise training may attenuate mitochondrial dysfunction in atorvastatin-treated skeletal muscles.

The present study had several limitations. Firstly, we did not include a CON+EXE group to better understand the effect of exercise on the ATO+EXE group. Secondly, we did not investigate the presence of a relevant mechanistic link between forelimb grip strength and skeletal muscle types in vitro. Finally, although mitochondrial H_2_O_2_ emission was significantly higher in the ATO group than in the CON group, this study did not show a direct link between mitochondrial H_2_O_2_ and mitochondrial damage. We acknowledge that further study is needed to demonstrate whether mitochondrial H_2_O_2_ emission affects mitochondrial damage or vice versa.

## 5. Conclusions

We have shown that long-term treatment with atorvastatin induced skeletal muscle myopathies such as increased CK level, muscle damage, reduced muscle strength, and impaired mitochondrial function. However, exercise training could attenuate myopathy and mitochondrial dysfunction caused by atorvastatin in rats. Therefore, these results provide evidence that exercise training may play a therapeutic role in regulating atorvastatin-induced muscle damage, muscle fatigue, and mitochondrial dysfunction in skeletal muscle, suggesting a novel strategy for patients using atorvastatin.
